# Supplementary material for: Self-anticoagulant sponge for whole blood auto-transfusion and its mechanism of coagulation factor inactivation
Source: Nat Commun. 2023 Aug 12;14:4875. doi: 10.1038/s41467-023-40646-7 (PMC10423252; doi:10.1038/s41467-023-40646-7)
Supplement: Supplementary file 1 — Supplementary Information [file 41467_2023_40646_MOESM1_ESM.pdf]

Supplementary Information

Self-anticoagulant sponge for whole blood auto-transfusion and its mechanism of coagulation factor inactivation

Tao Xu,[a] Haifeng Ji,[a,b] Lin Xu,[a] Shengjun Cheng,[a] Xianda Liu,[a] Yupei Li,[c] Rui Zhong,[d] Weifeng Zhao,[a] Jayachandran N. Kizhakkedathu,[b,e] and Changsheng Zhao.[a,f]

[a] *College of Polymer Science and Engineering, State Key Laboratory of Polymer Materials Engineering, Sichuan University, Chengdu, 610065, People's Republic of China*

[b] *Department of Pathology and Lab Medicine & Centre for Blood Research & Life Science Institute, University of British Columbia, 2350 Health Sciences Mall, Life Sciences Centre, Vancouver, BC, Canada V6T 1Z3*

[c] *Department of Nephrology, West China Hospital, Sichuan University, Chengdu, 610041, China*

[d] *Institute of Blood Transfusion, Chinese Academy of Medical Sciences, Peking Union Medical College, Chengdu 610052, China*

[e] *School of Biomedical Engineering, University of British Columbia, 2350 Health Sciences Mall, Life Sciences Centre, Vancouver, BC, Canada V6T 1Z3*

[f] *School of Chemical Engineering, Sichuan University, Chengdu, 610065, People's Republic of China*

Correspondence should be addressed to H.J. (E-mail: 903699293@qq.com) and W.Z. (E-mail: zhaoscukth@163.com).

Contents

Supplementary Figure 1. Digital photos of whole blood collected around the wound by vacuum tubes containing citrate sodium and EDTA 3
Supplementary Figure 2. ^{1}H NMR spectrum (DMSO-d6, 400 MHz) of DOPAm 4
Supplementary Figure 3. ^{13}C NMR spectrum (DMSO-d6, 100 MHz) of DOPAm 5
Supplementary Figure 4. Energy dispersive spectra of sponges 6
Supplementary Figure 5. X-ray photoelectron spectroscopy wide spectra of sponges 7
Supplementary Figure 6. Fourier transform infrared spectra of sponges 8
Supplementary Figure 7. Pore structure of sponges characterized by mercury intrusion porosimetry . 9
Supplementary Figure 8. Permeating behavior of one water droplet on the surface of sponges 10
Supplementary Figure 9. Schematic illustration of the customized setup for wicking method[1] 11
Supplementary Figure 10. Change of pH values of PBS after treatment by MS@D-HMP 12
Supplementary Figure 11. Typical compressive stress-strain curves of MS@D-HMP 13
Supplementary Figure 12. Thermogravimetric analysis of sponges 14
Supplementary Figure 13. Clotting times of PPP after incubation with MS and MS@D 15
Supplementary Figure 14. Calcium ions adsorption behaviors of MS@D-HMP in the solution of calcium ions and plasma 16
Supplementary Figure 15. Plasma recalcification times of MS@D-HMP at different concentrations 17
Supplementary Figure 16. Activities of FVIII, FIX, FXI and FXII in the plasma after incubation with MS and MS@D 18
Supplementary Figure 17. SDS-PAGE analysis of proteins at stage 1 and 2 19
Supplementary Figure 18. Proteomics analysis of tightly-bound proteins in stages 1 and 2 20
Supplementary Figure 19. Quantitative monitoring of FIXa in normal plasma 22
Supplementary Figure 20. Evaluation of FXIIa activity in the FXII solution after incubation with MS@D-HMP 23
Supplementary Figure 21. Effect of fibrinogen on the prolongation of TT for the plasma after incubation with MS@D-HMP. 24
Supplementary Figure 22. Leakage of coating from sponge and its effect on clotting time 25
Supplementary Figure 23. Effect of ATIII and HCII on the anticoagulant behaviors of sponge 26
Supplementary Figure 24. Correction of coagulation function by replenishing fresh normal plasma. 27
Supplementary Figure 25. Total numbers of collected blood cells after washing 28
Supplementary Figure 26. The result of blood count assay for the blood after incubation with the MS@D-HMP 29
Supplementary Figure 27. SEM pictures of the adhered platelet on sponges 30
Supplementary Figure 28. Flow cytometry gating strategy used to identify platelets and monocytes 31
Supplementary Figure 29. Clotting behaviors of blood collected using MS@D-HMP in the rabbit femoral artery hemorrhage model 32
Supplementary Figure 30. SEM pictures of the blood cells on the surface of MS@D-HMP in animal

experiments 33
Supplementary Figure 31. Survival of rabbits one month after the whole blood auto-transfusion 34
Supplementary Figure 32. Titration experiments *in vivo* for heparin-treated group and MS@D-HMP-treated group at different time intervals 35
Supplementary Figure 33. Clotting times *in vivo* for heparin-treated group and MS@D-HMP-treated group at different time intervals 36
Supplementary Table 1. Surface elemental compositions of sponges. 37
Supplementary Table 2. Calculated N/C ratio of sponges based on EDS and XPS analysis...............38
Supplementary Table 3. Elemental analysis of sponges before and after modification 39
Supplementary Table 4. Summary of physical properties of sponges 40
Supplementary Table 5. Change of calcium concentrations in MS@D-HMP-incubated plasma at different concentrations 41
Supplementary Table 6. Calcium ions adsorption behaviors of MS@D-HMP in plasma environment 42
Supplementary Table 7. Results of thromboelastography before and after incubation with MS@D-HMP 43
Supplementary Table 8. Detailed parameters for fluorescence-based FIXa activity assay 44
Supplementary Table 9. Results of blood count assay *in vitro* 45
Supplementary Table 10. Complete blood count assay for the transfused rabbit treated with heparin or MS@D-HMP at different time intervals 46
Supplementary References 47

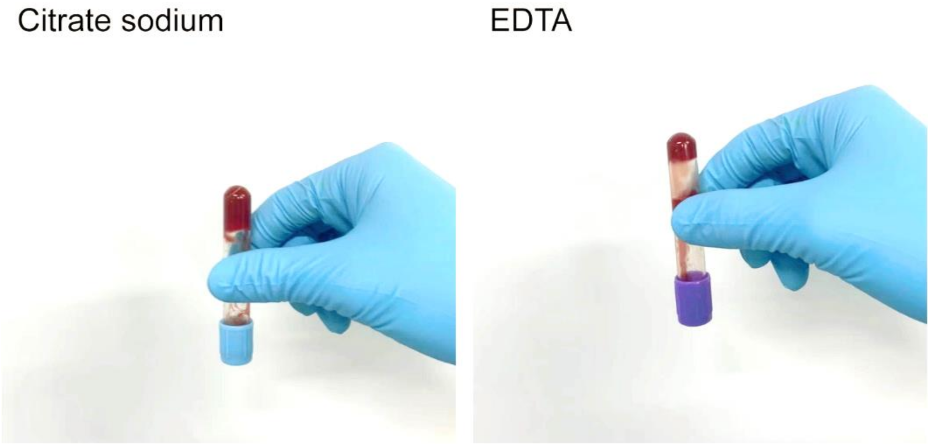

Supplementary Figure 1. Digital photos of whole blood collected around the wound by vacuum tubes containing citrate sodium and EDTA. A preliminary animal experiment in rabbit was conducted to explore the efficacy of calcium ions-chelating anticoagulants in the blood salvage. For the blood collected from the wound around femoral artery of rabbit, clot formation was observed within 3 min for both citrate- and EDTA-treated group (excessive anticoagulation was applied considering the higher serum calcium concentration of rabbit; ~1 mL whole blood was collected by using the standard 2-mL vacuum tubes).

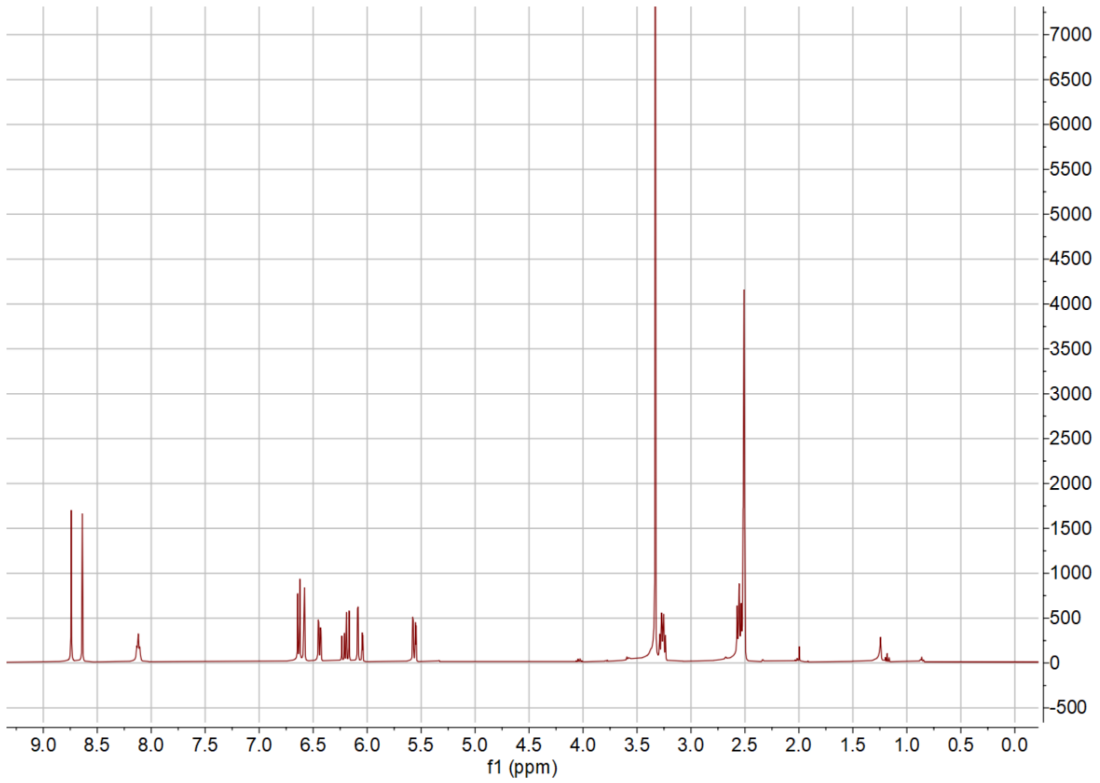

Supplementary Figure 2. ^{1}H NMR spectrum (DMSO-d6, 400 MHz) of DOPAm.

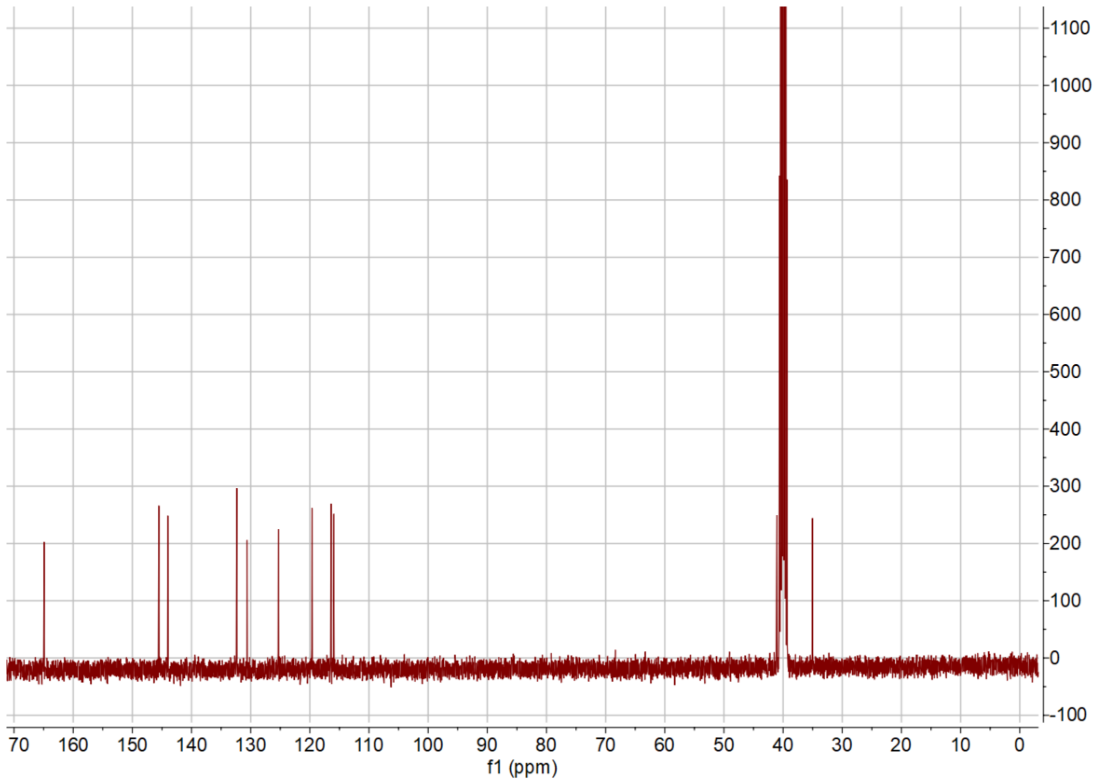

Supplementary Figure 3. ^{13}**C NMR spectrum (DMSO-d6, 100 MHz) of DOPAm.**

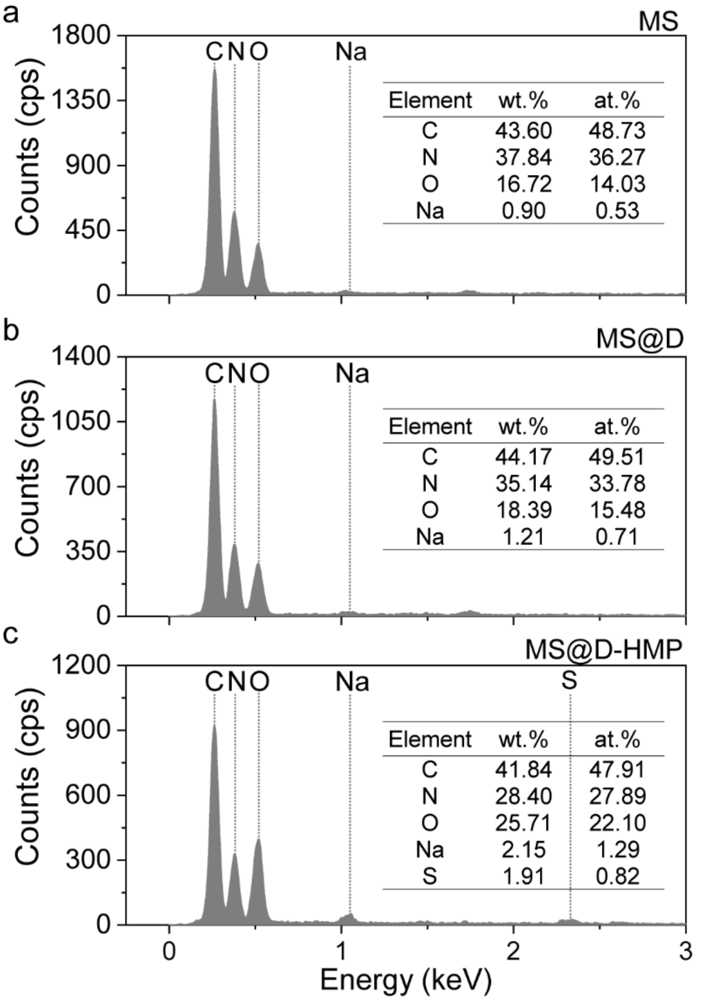

Supplementary Figure 4. Energy dispersive spectra of sponges. **a** MS. **b** MS@D. **c** MS@D-HMP. The mass fractions (wt.%) and atom fractions (at.%) of C, O, N, Na, and S elements in the sponges were shown in the inserted tables.

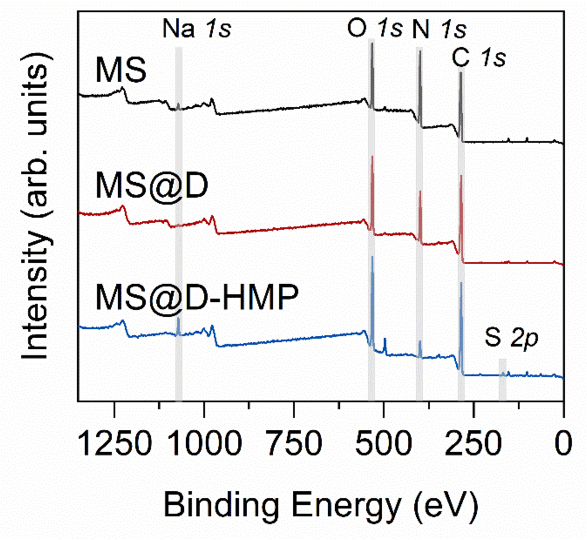

Supplementary Figure 5. X-ray photoelectron spectroscopy wide spectra of sponges.

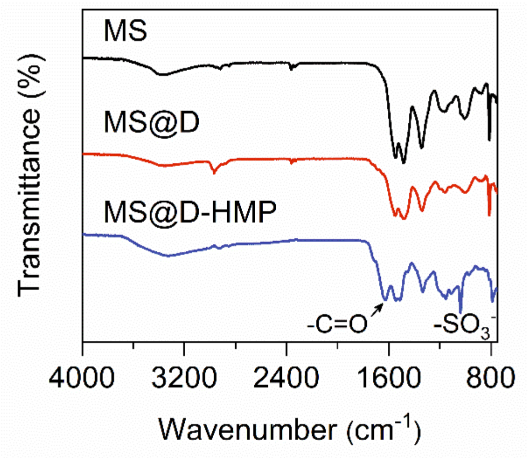

Supplementary Figure 6. Fourier transform infrared spectra of sponges. The repetition times were determined by the scanning times of FTIR equipment.

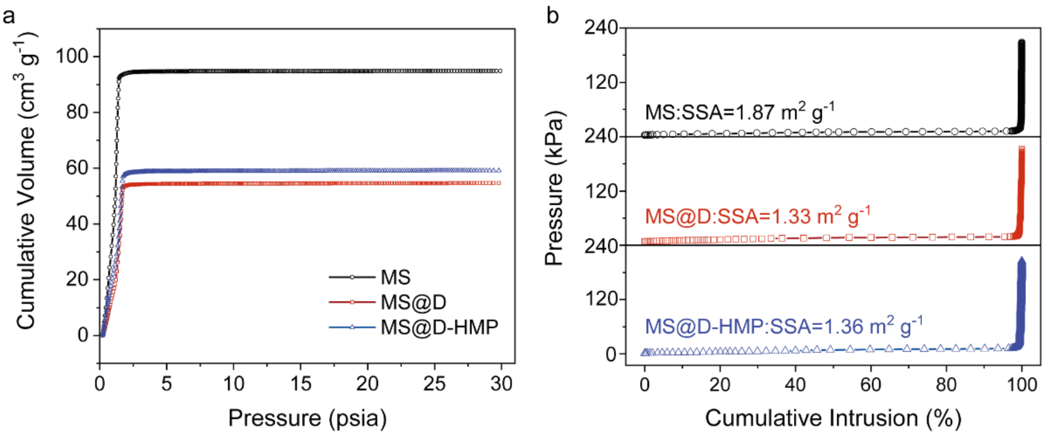

Supplementary Figure 7. Pore structure of sponges characterized by mercury intrusion porosimetry. a Raw mercury intrusion curves of sponges. **b** Pressure-cumulative mercury intrusion percentage curves of sponges.

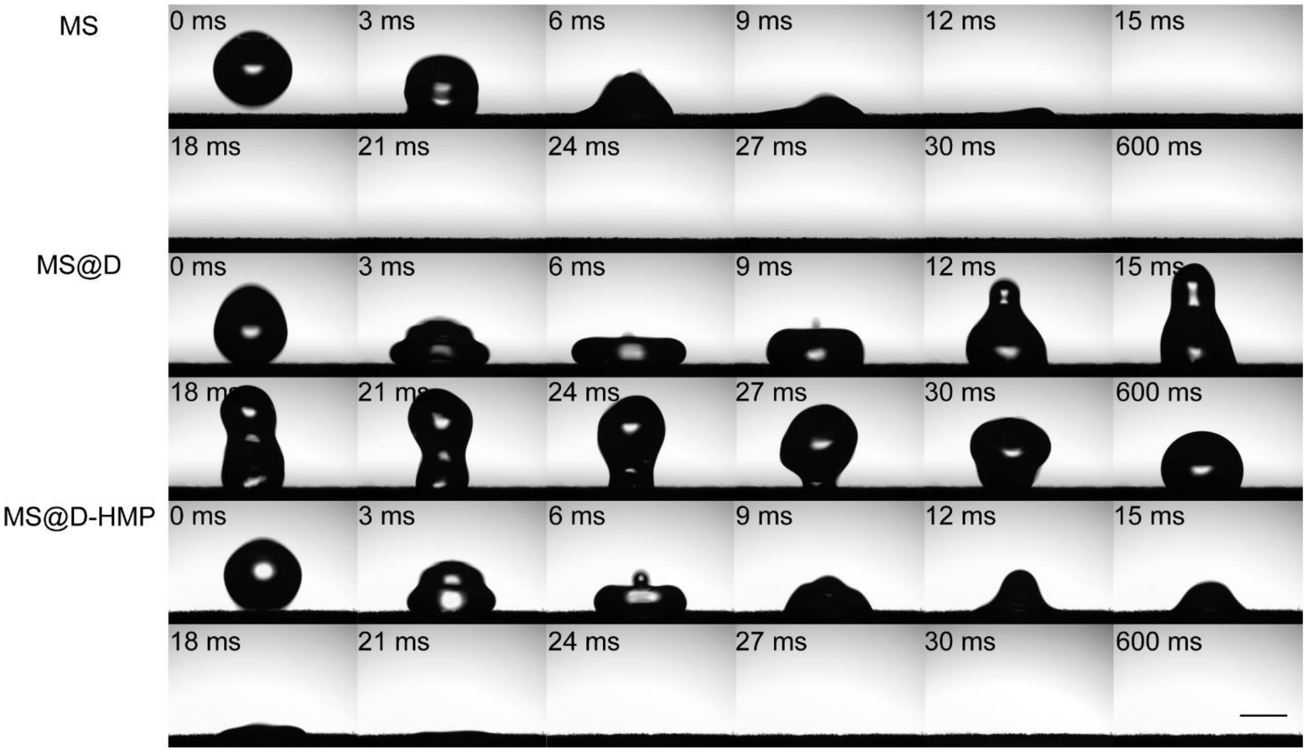

Supplementary Figure 8. Permeating behavior of one water droplet on the surface of sponges. Snapshots showed the impact dynamics of a 20 μL droplet on the surface of sponges. For MS, the droplet immediately spread on the surface of MS after impacting and deposited on the surface. MS@D was endowed with hydrophobic property due to the deposition of vinyl-containing DOPAm. For MS@D-HMP, the droplet impacting the surface of MS@D-HMP first rebounded and then finally deposited on the surface. Scale bars, 2 mm.

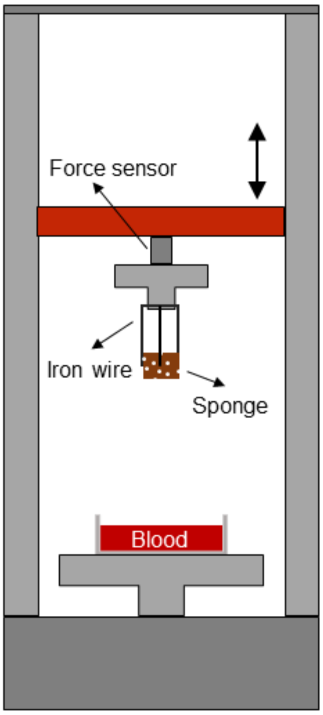

Supplementary Figure 9. Schematic illustration of the customized setup for wicking method[1]. A sponge monolith (3 cm × 3 cm × 1.5 cm) was fixed below the movable force sensor of a universal mechanical testing machine (SANS CMT4000) by iron wires, which could avoid the sorbents tilting during the liquid-absorbing process. Specific liquid (deionized water or heparinized goat whole blood) was put on a vertical stage. In a typical measurement process, the force sensor was gradually moved down at a speed of 10 mm/min to get the liquid surface contacted with the bottom surface of MS@D-HMP. Once a force above 0.001 N was detected, the force sensor was stopped and fixed at this position for 60 s until sorption was saturated. Then, the force sensor was gradually lifted up at a speed of 10 mm/min to separate the bottom of sponge from liquid surface. The real liquid absorption kinetics were obtained via eliminating the effect of surface tension[2]. Finally, K_s could be derived from the slope of linear relation between the mass of liquid absorbed per unit area (m_s) and the square root of sorption time ($t_{1/2}$).

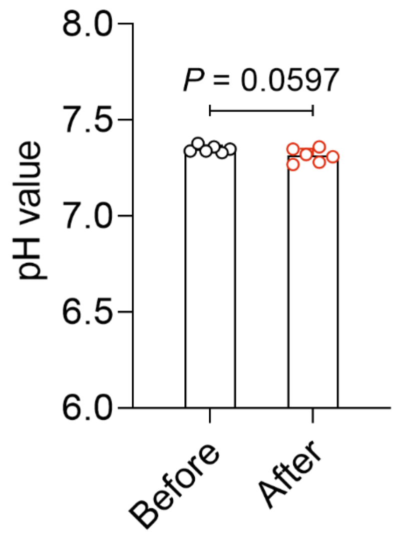

Supplementary Figure 10. Change of pH values of PBS after treatment by MS@D-HMP. MS@D-HMP exhibited no significant influence on the pH value of PBS after treatment, indicating the successful deprotonation of HMP (n = 3 biologically independent samples, mean ± SD. Unpaired, two-tailed student's *t*-test)

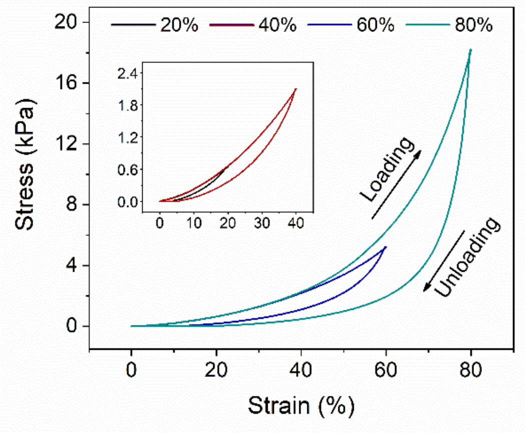

Supplementary Figure 11. Typical compressive stress-strain curves of MS@D-HMP. The experiments were performed independently in duplicate with similar results.

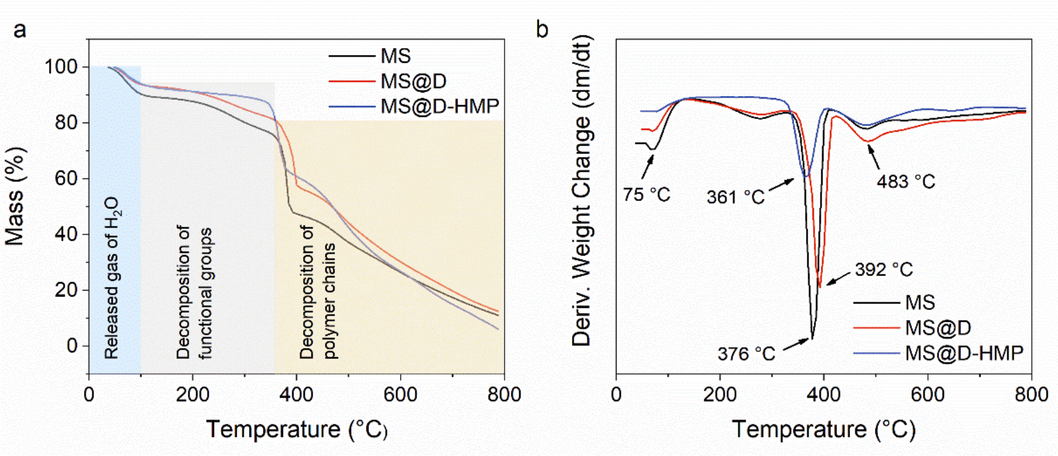

Supplementary Figure 12. Thermogravimetric analysis of sponges. **a-b** TGA curves (**a**) and DTG curves (**b**) of MS, MS@D and MS@D-HMP. The peaks at 376 °C in the curve of MS shifted to 392 and 361 °C in those of MS@D and MS@D-HMP, respectively. The experiments were performed independently in duplicate with similar results.

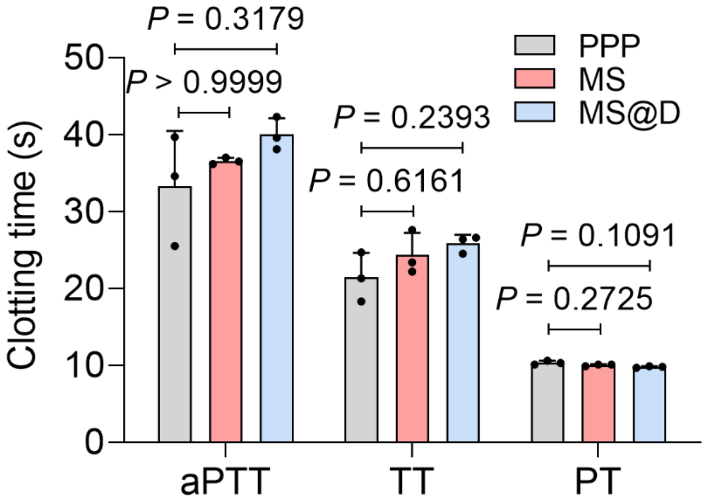

Supplementary Figure 13. Clotting times of PPP after incubation with MS and MS@D. The clotting times of plasma after incubation with MS and MS@D were similar to the control group, indicating the anticoagulant ability of MS@D-HMP was mainly ascribed to the coated HMP (n = 3 biologically independent samples, mean ± SD. One-way ANOVA with Bonferroni post-hoc tests).

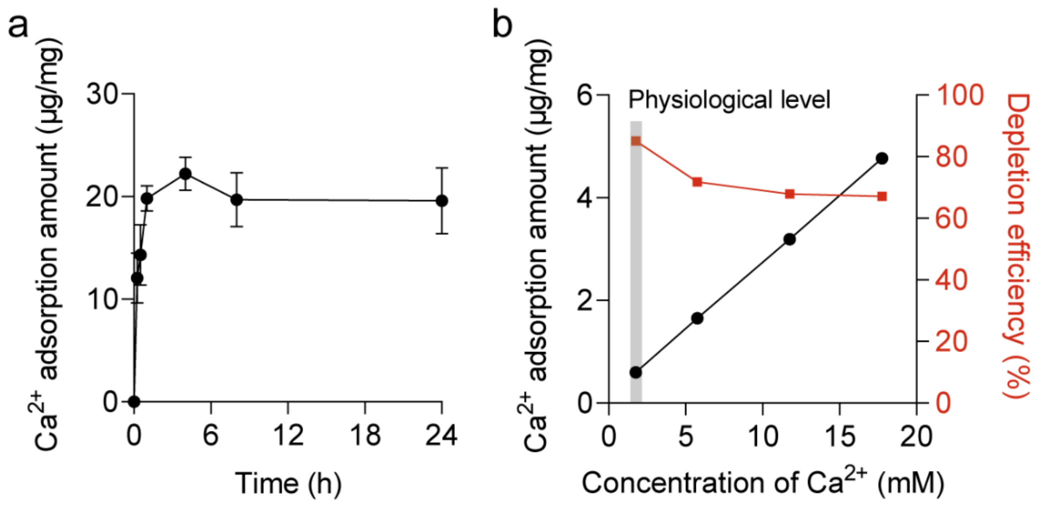

Supplementary Figure 14. Calcium ions adsorption behaviors of MS@D-HMP in the solution of calcium ions and plasma. **a** Calcium ions adsorption amount of MS@D-HMP in calcium ions solution (2 mM) at different incubation times (n = 3 independent samples, mean ± SD). **b** Equilibrium binding capacity and the corresponding depletion efficiency of calcium ions by MS@D-HMP in hirudin-anticoagulated plasma environment (n = 3 biologically independent samples, mean ± SD).

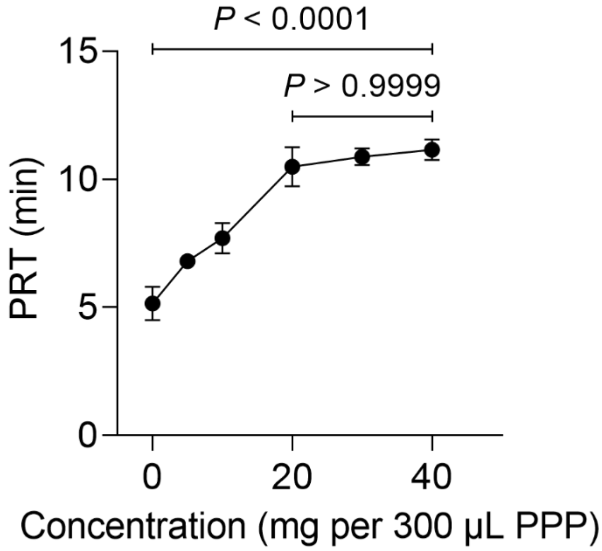

Supplementary Figure 15. Plasma recalcification times of MS@D-HMP at different concentrations. MS@D-HMP was taken out before recalcification to exclude the contribution of adsorption of calcium ions by sponges on the prolonged PRT (n = 3 biologically independent samples, mean ± SD. One-way ANOVA with Bonferroni post-hoc tests).

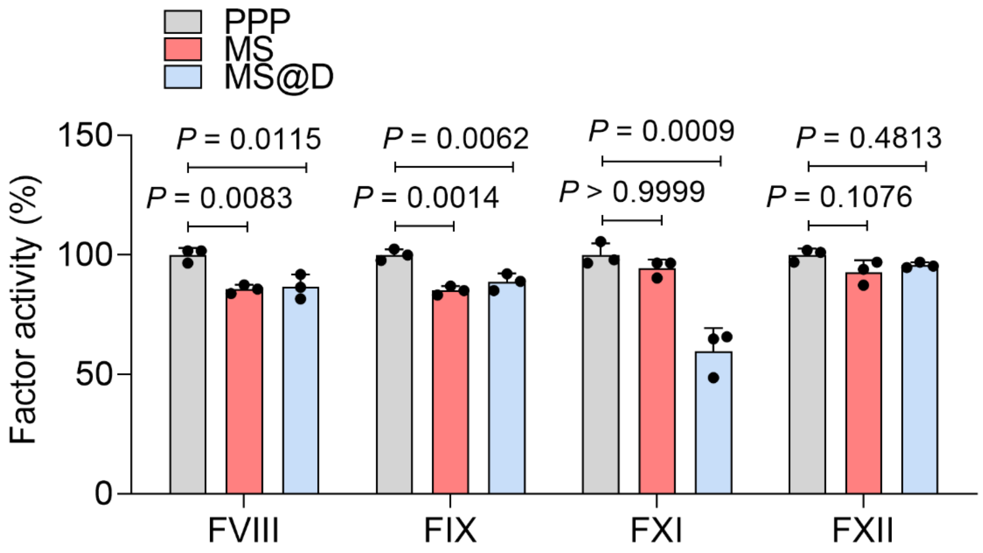

Supplementary Figure 16. Activities of FVIII, FIX, FXI and FXII in the plasma after incubation with MS and MS@D. Comparing with PPP, the activities of FVIII, FIX and FXI decreased a little (n = 3 biologically independent samples, mean ± SD. One-way ANOVA with Bonferroni post-hoc tests).

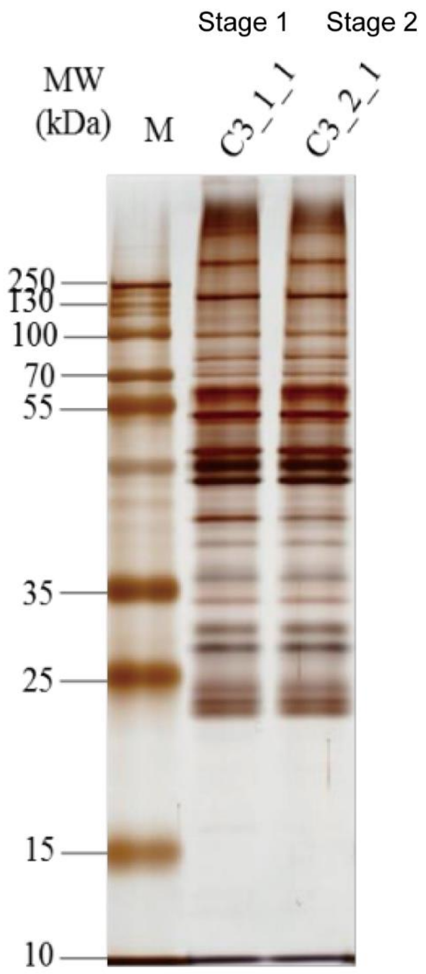

Supplementary Figure 17. SDS-PAGE analysis of proteins at stage 1 and 2. Similar bands of proteins were observed at different stages. The experiment was performed once.

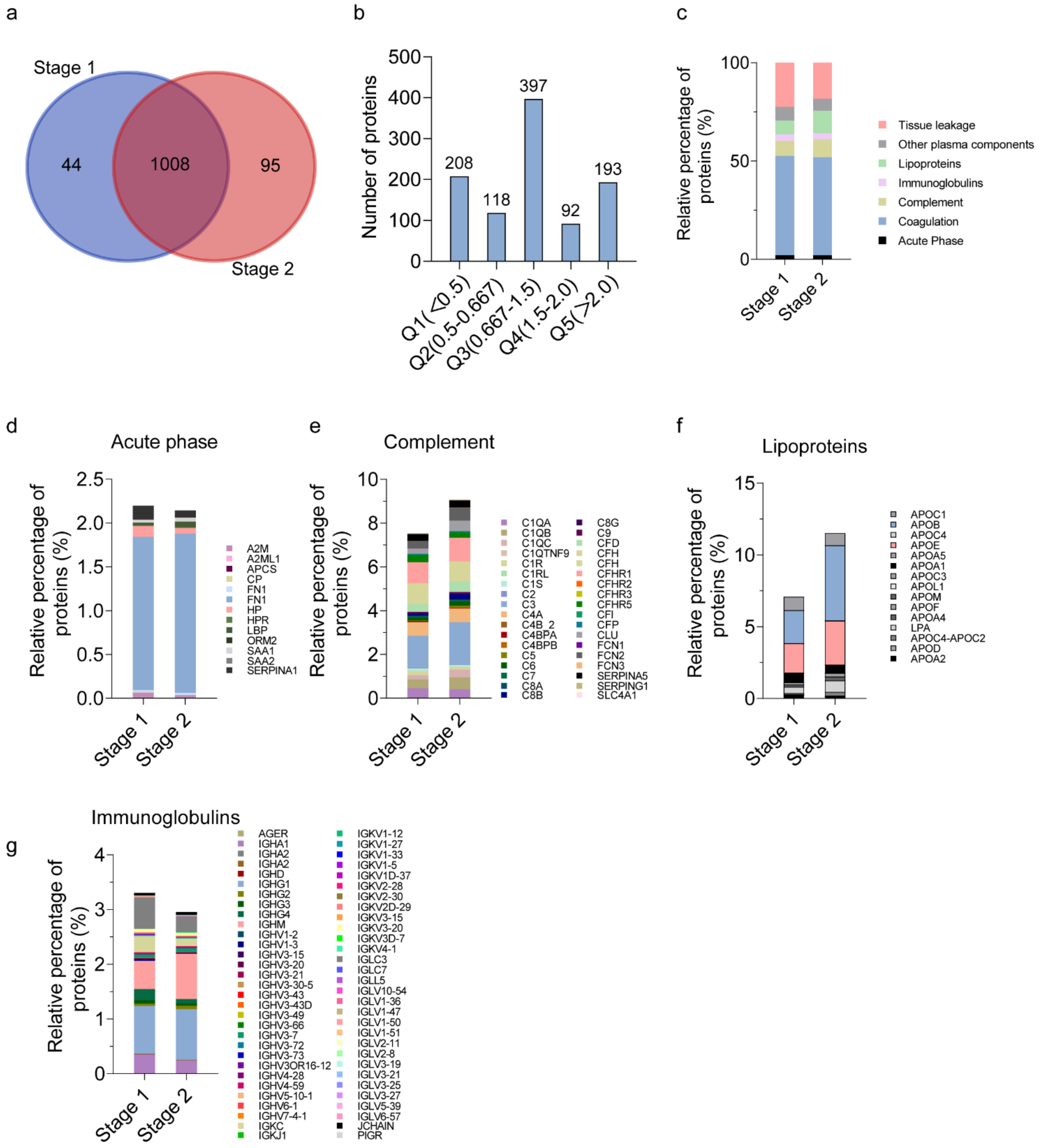

Supplementary Figure 18. Proteomics analysis of tightly-bound proteins in stages 1 and 2. a Unique and common proteins between stage 1 and 2 depicted as a Venn diagram. **b** Summary on fold change (Q) for relative abundance of different proteins at stage 1 and 2. Q was determined by relative abundance of proteins at stage 1 over that at stage 2: Q1 (< 0.5); Q2 (0.5-0.667); Q3 (0.667-1.5); Q4 (1.5-2.0) and Q5 (>2.0). **c** Classification of tightly-bound proteins in stages 1 and 2 based on biological function. Values are expressed in relative percentages based on total proteins. Proteins in stage 1 was composed in the order of coagulation proteins, tissue leakage proteins, complement proteins, lipoproteins, other plasma components, immunoglobulins and acute phase reactants. In stage 2, the complement proteins and lipoproteins were increased, while the acute phase reactants, coagulation proteins, immunoglobulins, other plasma components and tissue leakage were decreased. **d-g**

Bioinformatic classification of tightly-bound proteins in each biological group in stages 1 and 2: acute phase (**d**), complement proteins (**e**), lipoproteins (**f**) and immunoglobulins (**g**). Values are expressed in relative percentages based on total proteins.

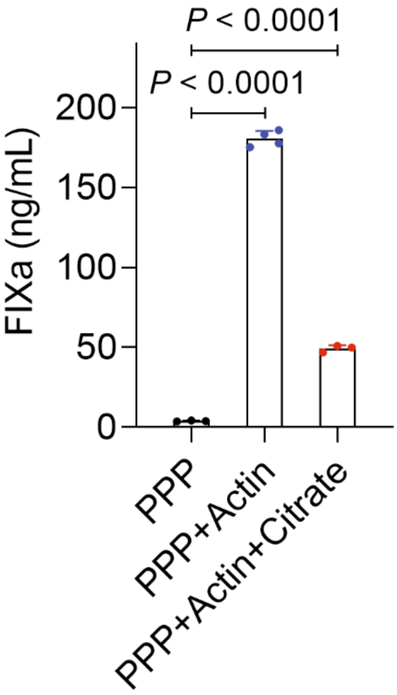

Supplementary Figure 19. Quantitative monitoring of FIXa in normal plasma. Citrate was added to block the activation of FIX to FIXa by FXIa. The citrate could only chelate the calcium ions in the plasma (1:9 v/v), and the calcium ions supplemented in the assay kit was sufficient for FIXa to proteolytically activate factor X (n = 3 biologically independent samples for PPP and PPP+Actin+Citrate, n = 4 biologically independent samples for PPP+Actin, mean ± SD. One-way ANOVA with Bonferroni post-hoc tests).

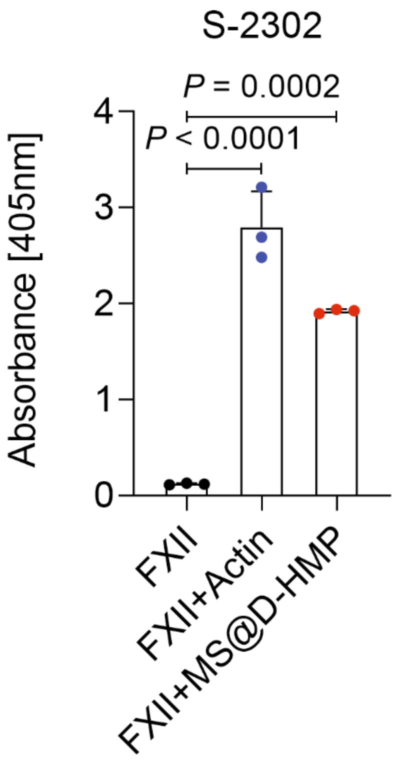

Supplementary Figure 20. Evaluation of FXIIa activity in the FXII solution after incubation with MS@D-HMP. Hydrolysis of the chromogenic FXIIa substrate S-2302 was detected at an absorbance = 405 nm (n = 3 biologically independent samples, mean ± SD. One-way ANOVA with Bonferroni post-hoc tests)

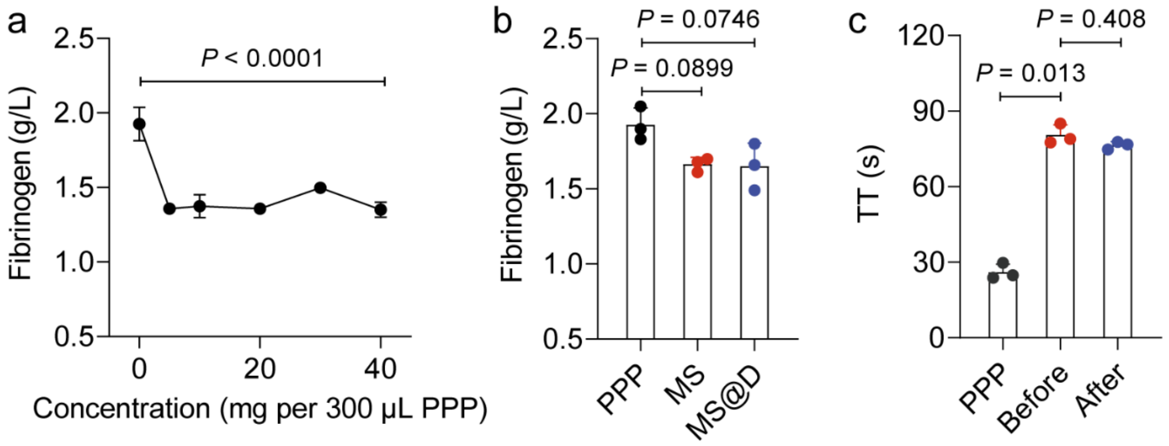

Supplementary Figure 21. Effect of fibrinogen on the prolongation of TT for the plasma after incubation with MS@D-HMP. a Concentration of fibrinogen in the PPP after incubation with MS@D-HMP at different concentrations (n = 3 biologically independent samples, mean ± SD. One-way ANOVA with Bonferroni post-hoc tests). **b** Concentration of fibrinogen in the PPP after incubation with MS and MS@D at a concentration of 30 mg per 300 µL PPP (n = 3 biologically independent samples, mean ± SD. One-way ANOVA with Bonferroni post-hoc tests). **c** TT correction assay via replenishing fibrinogen. After replenishing the fibrinogen in the MS@D-HMP-incubated plasma to normal range, the TT values exhibited no significant change compared to the group without addition of fibrinogen. The result indicated that adsorption of fibrinogen contributed little to the prolongation of TT values in the MS@D-HMP-incubated plasma (n = 3 biologically independent samples, mean ± SD. One-way ANOVA with Bonferroni post-hoc tests).

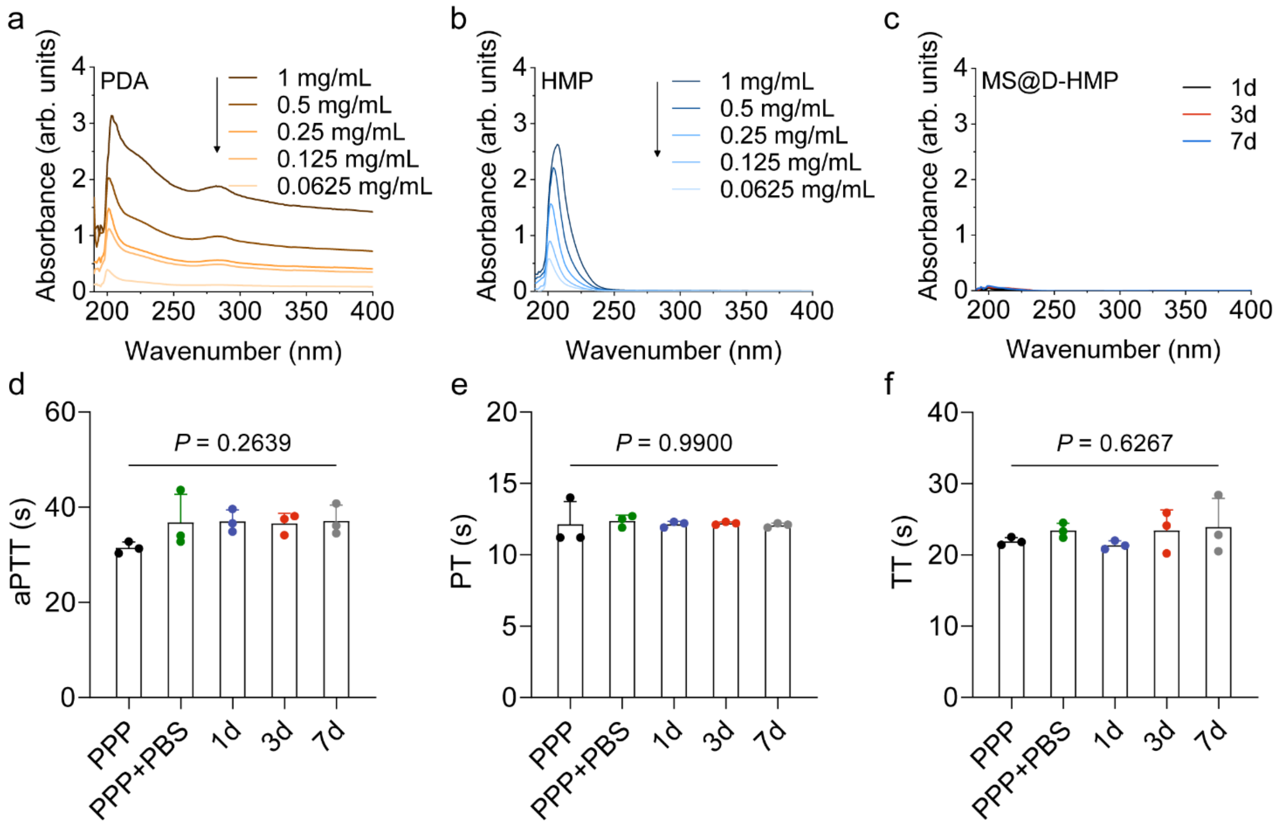

Supplementary Figure 22. Leakage of coating from sponge and its effect on clotting time. **a-c** UV-vis spectra for gradient-diluted polydopamine (**a**), HMP (**b**), and the eluate after incubation for 1 day, 3 days and 7 days (**c**). **d-f** Clotting times including aPTT (**d**), PT (**e**), and TT (**f**) of PPP mixed with the eluate after incubation for 1 day, 3 days and 7 days (n = 3 biologically independent samples, mean ± SD. One-way ANOVA with Bonferroni post-hoc tests).

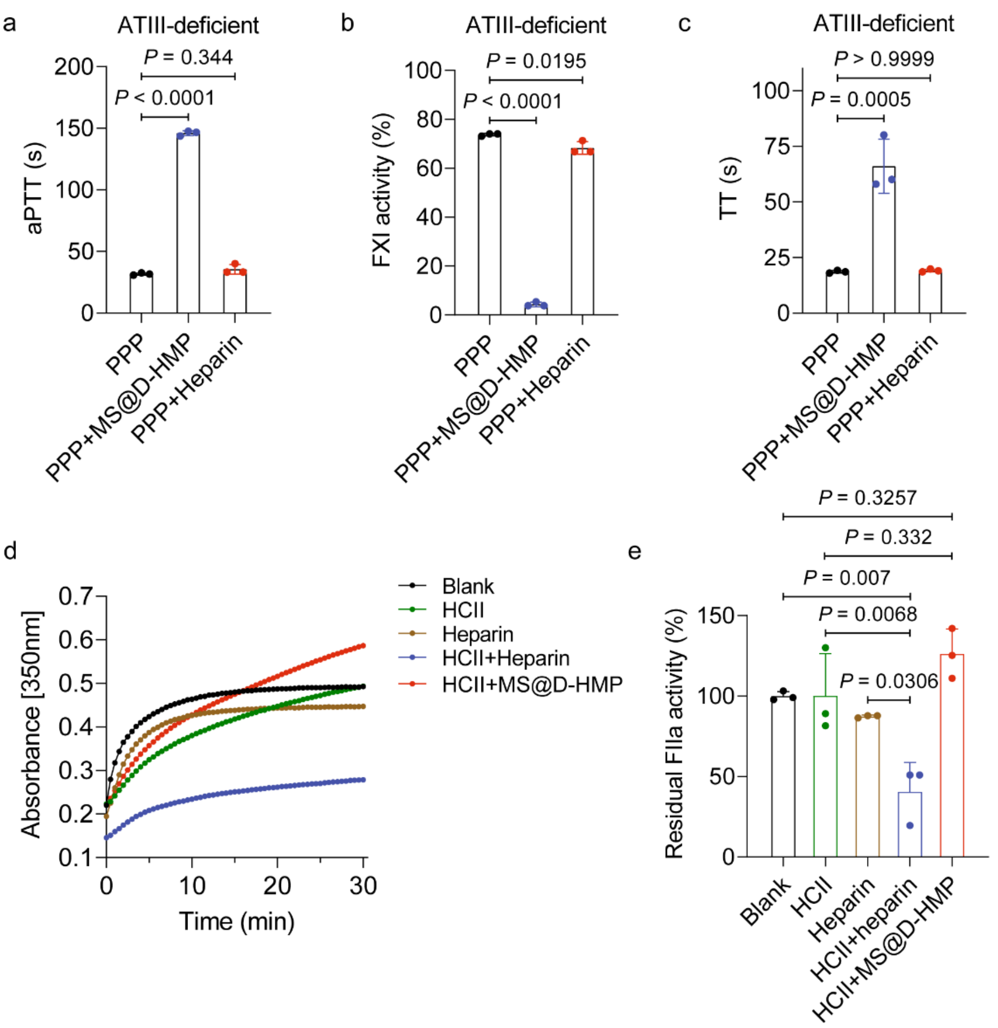

Supplementary Figure 23. Effect of ATIII and HCII on the anticoagulant behaviors of sponge. **a**-**c** aPTT values (**a**), FXI activity (**b**) and TT values (**c**) of ATIII-deficient plasma after incubation with MS@D-HMP. Heparin-anticoagulated ATIII-deficient plasma (0.1 IU/100 μL ATIII-deficient plasma) was used for comparison (n = 3 biologically independent samples, mean ± SD. One-way ANOVA with Bonferroni post-hoc tests). **d** Effect of MS@D-HMP on the inhibition of FIIa-mediated fibrin polymerization in the presence of HCII. Turbidity change was monitored by absorbance at 350 nm. (n = 3 biologically independent samples, mean ± SD). **e** Calculated residual FIIa activity in the presence of HCII (n = 3 biologically independent samples, mean ± SD. One-way ANOVA with Bonferroni post-hoc tests).

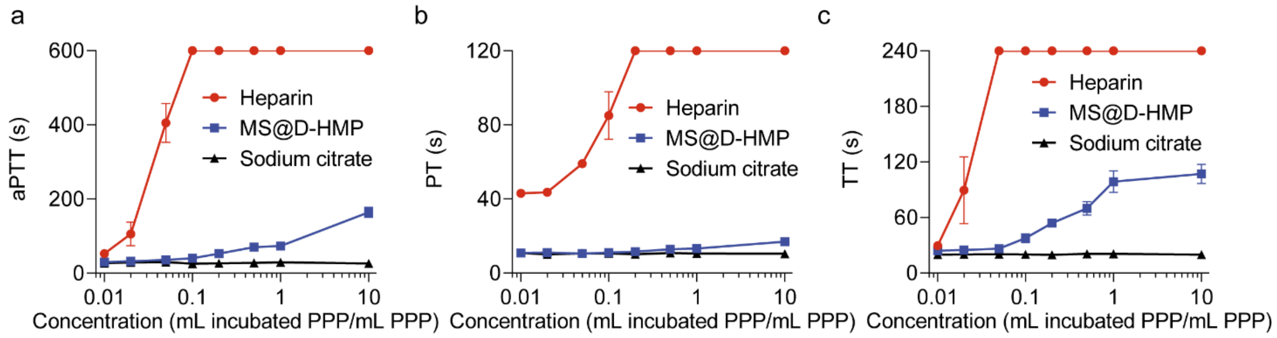

Supplementary Figure 24. Correction of coagulation function by replenishing fresh normal plasma. a-c TT (**a**), PT (**b**) and aPTT (**c**) of different sample plasma after mixing with fresh citrate-anticoagulated normal plasma at different concentrations. Citrate-anticoagulated blood was collected using vacuum tubes (5 mL, Jiangsu Kangjian Inc., China) containing 3.8% sodium citrate with anticoagulant/blood ratio of 1:9. Heparin-anticoagulated blood was collected using commercial heparin vacuum tubes (5 mL, Jiangsu Kangjian Inc., China), and the final concentration of heparin was around 10 IU/mL blood. For plasma collection, the blood was centrifuged at 4000 rpm for 15 min to obtain PPP. For MS@D-HMP-incubated plasma, 10 mg MS@D-HMP was incubated with 100 µL plasma for 30 min, and the plasma was collected (n = 3 biologically independent samples, mean ± SD).

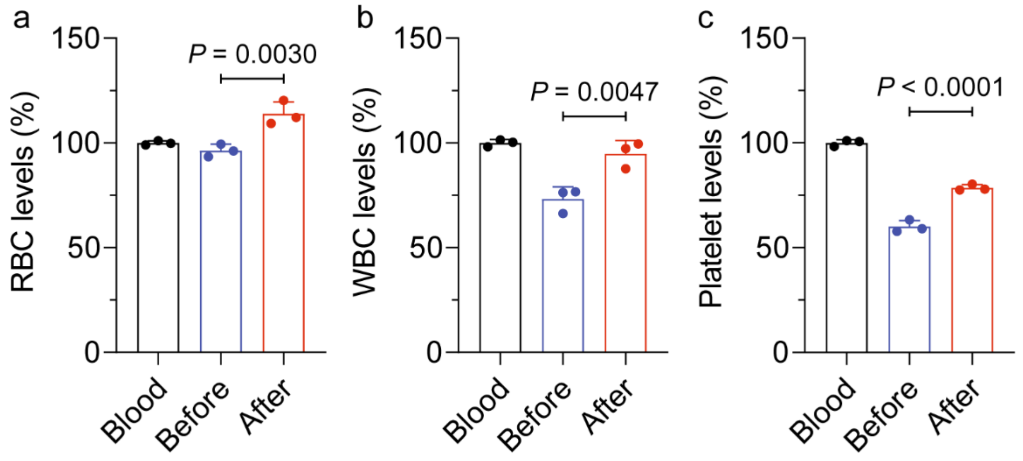

Supplementary Figure 25. Total numbers of collected blood cells after washing. **a-c** Blood cell levels including RBC (**a**), WBC (**b**) and platelet (**c**) before and after washing (n = 3 biologically independent samples, mean ± SD. One-way ANOVA with Bonferroni post-hoc tests).

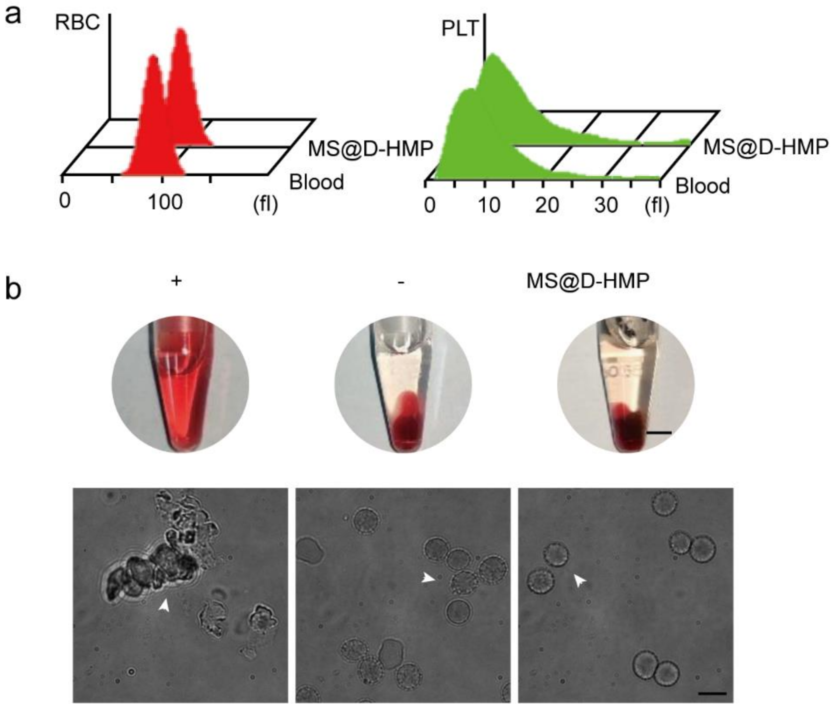

Supplementary Figure 26. The result of blood count assay for the blood after incubation with the MS@D-HMP. **a** Volume distribution curves of RBC (left) and platelet (right) for pristine whole blood and the MS@D-HMP-treated blood. **b** Digital photos of supernatants (upper) obtained by centrifugation of the salvaged blood (Scale bars, 5 mm), and the corresponding morphologies (lower) of RBCs (Scale bars, 10 μm). From left to right: DI water (+), PBS (-) and RBCs treated by the MS@D-HMP.

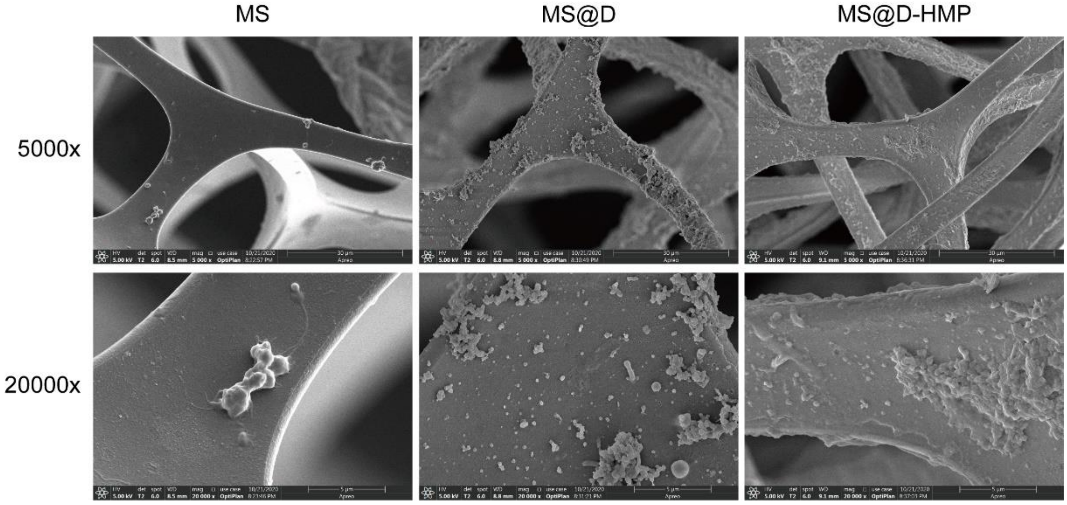

Supplementary Figure 27. SEM pictures of the adhered platelet on sponges. All the groups exhibited few platelets (~3 μm) adhered on the surface. For MS@D and MS@D-HMP, the micrometer-sized aggregated clusters (~1 μm) were attributed to the deposited PDA nanoparticles. The experiments were performed independently in duplicate with similar results.

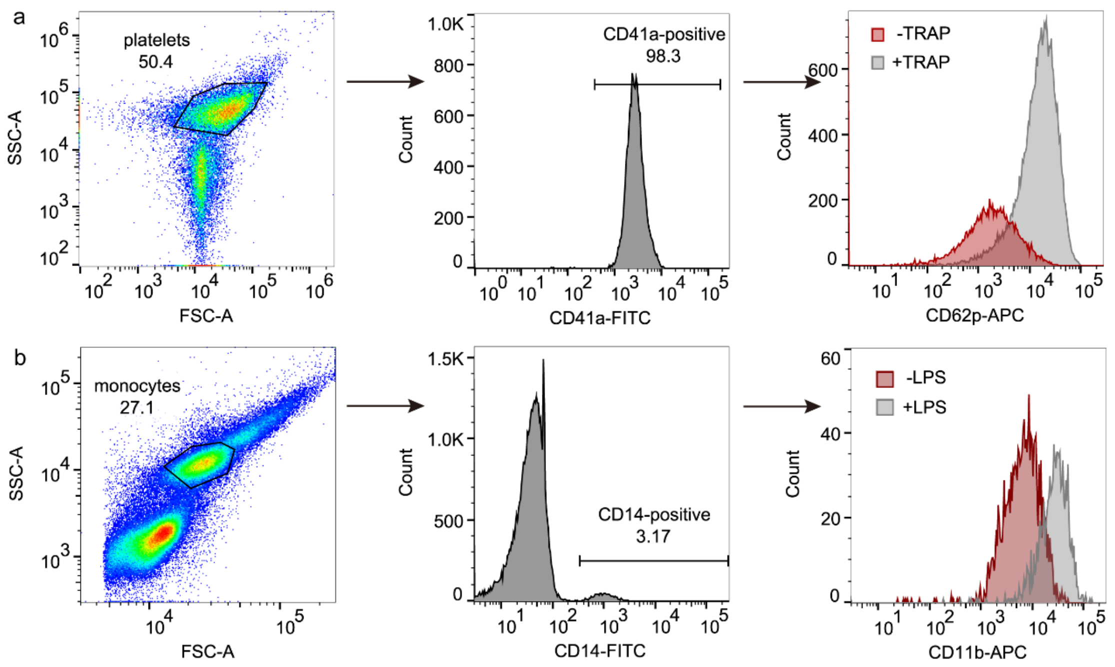

Supplementary Figure 28. Flow cytometry gating strategy used to identify platelets and monocytes. **a** Platelets were gated according to their characteristic appearance in the forward vs. side scatter plot and anti-CD41a-FITC (eBioscience™, #11-0419-42) antibody. Platelet activation was measured by measuring the expression of activation marker CD62p using anti-CD62p-APC (eBioscience™, #17-0626-82) antibody. **b** Monocytes were gated according to their characteristic appearance in the forward vs. side scatter plot and anti-CD14-FITC (eBioscience™, #11-0149-42) antibody. Monocyte activation was measured by measuring the expression of activation marker CD11b using anti-CD11b-APC (eBioscience™, #17-0118-42) antibody.

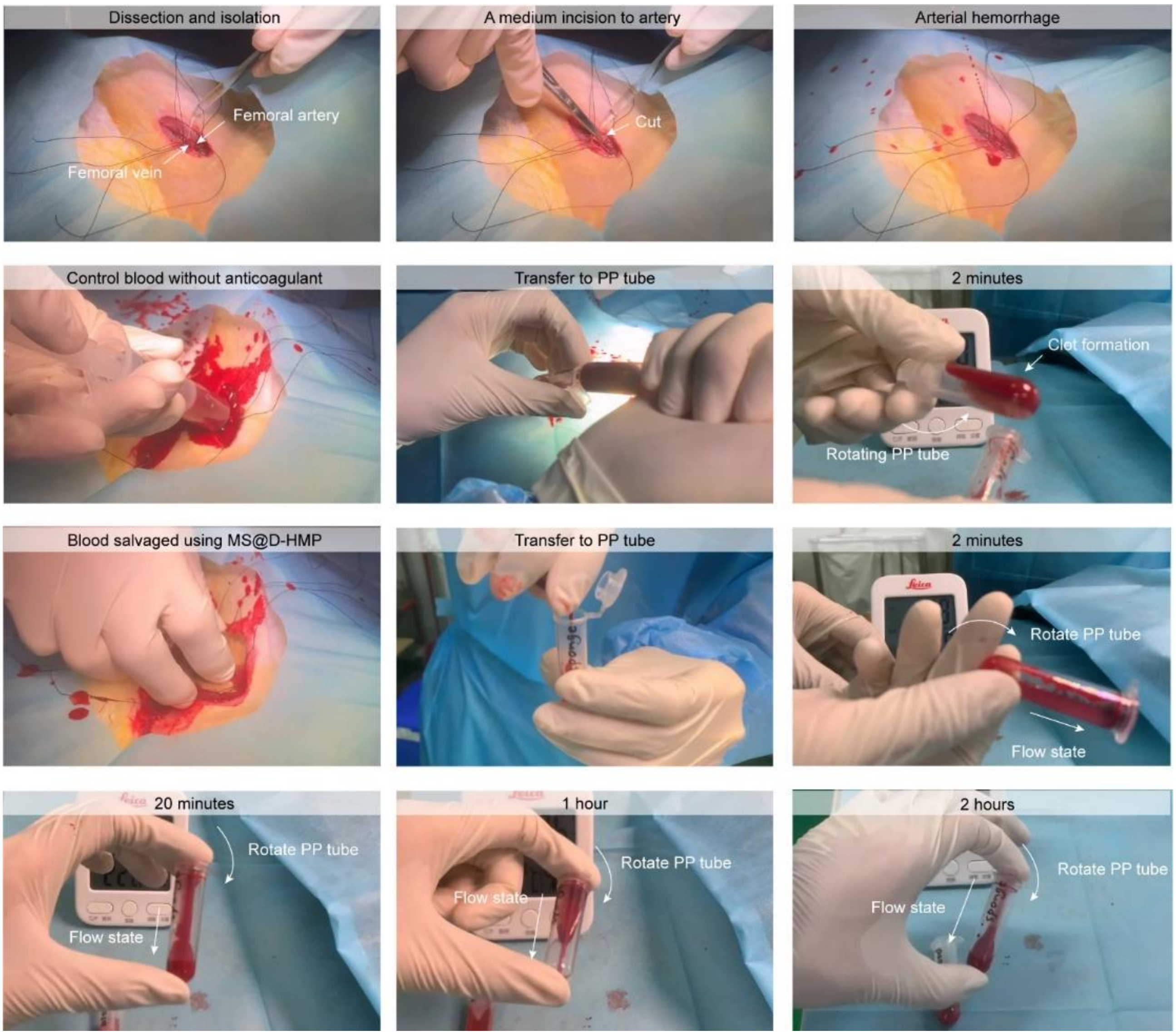

Supplementary Figure 29. Clotting behaviors of blood collected using MS@D-HMP in the rabbit femoral artery hemorrhage model. To compare the clotting times, control whole blood near the wound was also collected without anticoagulant using through a 5-mL syringe. It is obvious that clot formed in the control blood within 5 min, while blood collected using MS@D-HMP remained non-clotted for over 1 h.

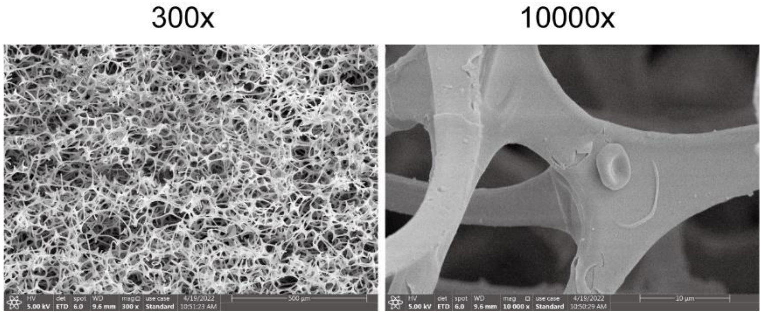

Supplementary Figure 30. SEM pictures of the blood cells on the surface of MS@D-HMP in animal experiments. No blood clot was observed, suggesting that MS@D-HMP could suppress the clot formation when recovering the blood. The adhered RBCs were few and retained their typical cell morphologies. The experiments were performed independently in duplicate with similar results.

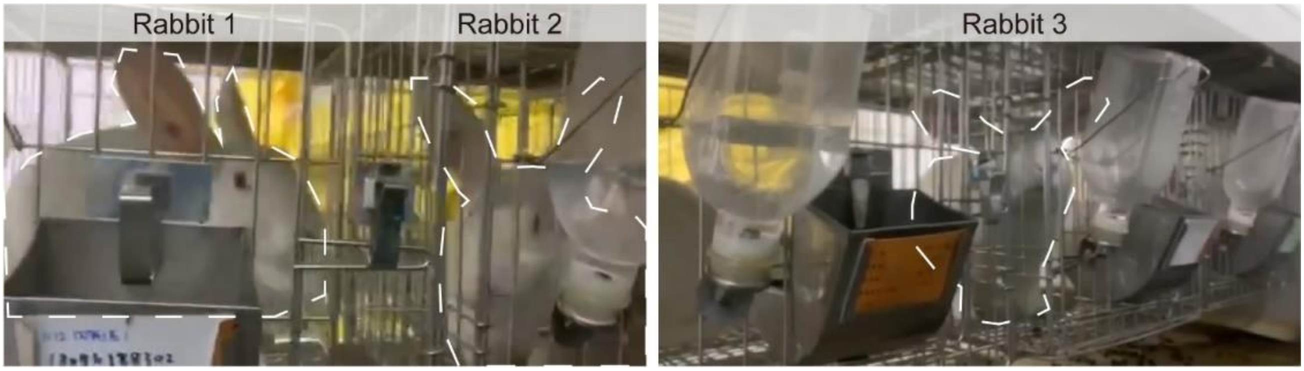

Supplementary Figure 31. Survival of rabbits one month after the whole blood auto-transfusion.

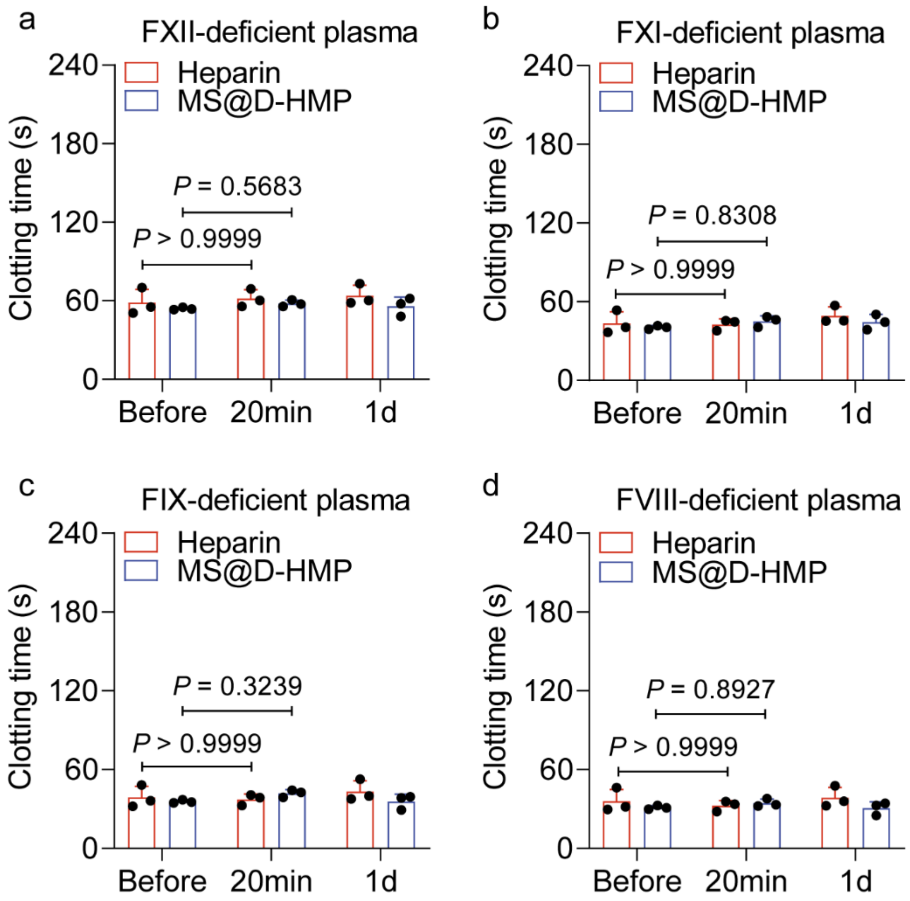

Supplementary Figure 32. Titration experiments *in vivo* for heparin-treated group and MS@D-HMP-treated group at different time intervals. **a** FXII-deficient plasma. **b** FXI-deficient plasma. **c** FIX-deficient plasma. **d** FVIII-deficient plasma (n = 3 biologically independent samples, mean ± SD. Two-way ANOVA with Geisser-Greenhouse correction and Bonferroni post-hoc tests).

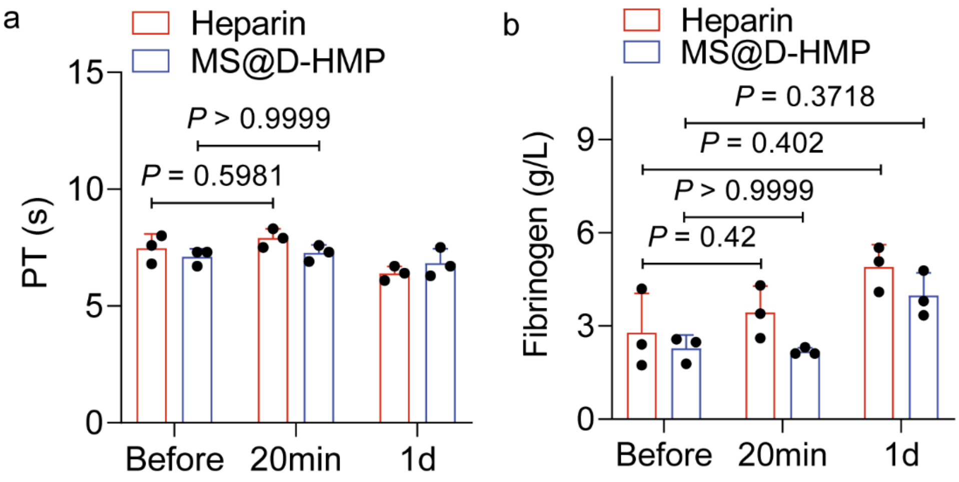

Supplementary Figure 33. Clotting times *in vivo* for heparin-treated group and MS@D-HMP-treated group at different time intervals. **a** PT. **b** Fibrinogen (n = 3 biologically independent samples, mean ± SD. Two-way ANOVA with Geisser-Greenhouse correction and Bonferroni post-hoc tests).

Supplementary Table 1. Surface elemental compositions of sponges. Data were obtained from XPS analysis.

Sample	C		N		O		S		Na	
	at.%	wt.%	at.%	wt.%	at.%	wt.%	at.%	wt.%	at.%	wt.%
MS	55.96	50.80	28.80	30.49	14.68	17.76	0	0	0.55	0.96
MS@D	63.20	57.96	20.40	21.82	16.07	19.64	0	0	0.33	0.58
MS@D-HMP	64.73	57.17	7.53	7.76	24.45	28.76	1.14	2.69	2.14	3.62

Supplementary Table 2. Calculated N/C ratio of sponges based on EDS and XPS analysis. The theoretical values of N/C ratio for DOPAm, DOPA and heparin-mimetic polymer were 1/13, 1/8 and 0.045, respectively[3]. The N/C ratio decreased with the introduction of DOPAm, AA and AMPS due to the high content of C in the DOPAm, AA and AMPS.

**Sample**	**EDS**	**XPS**
	**N/C (at.%/at.%)**	**N/C (at.%/at.%)**
MS	0.74	0.51
MS@D	0.68	0.32
MS@D-HMP	0.58	0.12

Supplementary Table 3. Elemental analysis of sponges before and after modification. PDA and HMP were used to calculate the compositions of MS@D-HMP. The deposited PDA calculated from N/C and N/H were 1.39% and 1.06%, respectively. The deposited HMP calculated from N/C and N/H were 1.73% and 2.86%, respectively.

Sample	**N (%)**	**C (%)**	**H (%)**
PDA	5.98	55.15	5.28
HMP	2.74	42.13	7.25
MS	39.54	31.38	4.93
MS@D	41.8	33.60	5.26
MS@D-HMP	39.5	32.28	5.11

Supplementary Table 4. Summary of physical properties of sponges.

Sample	Pore size (μm)		Total pore volume (cm^3 g^{-1})	Specific surface area (m^2/g)	Porosity (%)
	Mode	Mean			
MS	162.7	203.1	94.9	1.87	93.0
MS@D	138.0	163.7	54.6	1.33	77.6
MS@D-HMP	134.7	173.8	59.1	1.36	67.5

Supplementary Table 5. Change of calcium concentrations in MS@D-HMP-incubated plasma at different concentrations. Fresh PPP was was re-calcified $CaCl_2$ solution to obtain a final concentration at 12.5 mM. The re-calcified PPP was added into different concentration of sponges, and taken out (within 5 s) immediately. The calcium concentration before and after treatment was determined. All the values are expressed as mean ± SD, n = 3 biologically independent samples.

	**Fresh PPP**	**Concentration of the MS@D-HMP (mg per 300 µL PPP)**					
		**0**	**5**	**10**	**20**	**30**	**40**
Calcium concentrations (mmol/L)	1.98 ±0.02	12.90 ±0.67	9.96 ±1.01	8.40 ±0.61	5.13 ±0.11	4.45 ±0.49	5.69 ±0.92

Supplementary Table 6. Calcium ions adsorption behaviors of MS@D-HMP in plasma environment. Certain amounts of calcium ions were added into 400 μL hirudin anticoagulant plasma. The re-calcified plasma was incubated with 10 mg MS@D-HMP for 10 min.

Calcium supplementation dose (mM)	Serum calcium after supplementation (mM	Serum calcium after incubation with sponge (mM)
0	1.75	0.26
5	6.75	1.62
10	11.75	3.77
15	16.75	5.83

Supplementary Table 7. Results of thromboelastography before and after incubation with MS@D-HMP.

Items	Blood	MS@D-HMP	MS@D-HMP+Ca^{2+}
R (min)	10.8	> 80	22.9
K (min)	9.0	NA	57.6
α (deg)	24.6	NA	4.9
MA (mm)	32.6	NA	2.0
LY30 (%)	0.7	NA	0

Supplementary Table 8. Detailed parameters for fluorescence-based FIXa activity assay. The below components were mixed and incubated for 10 min. For the groups with sponges, the plasma was collected. The generation of FIXa in the incubated plasma was determined using a Fluorescence-based Factor IXa Activity Assay Kit (BioVision Inc., USA).

**Group**	**PPP[a] /dFXII PPP[b]**	**Ca^{2+} (50 mM)**	**Sodium citrate**	**MS@D-HMP**	**Actin**	**Saline**
	**(μL)**	**(μL)**	**(μL)**	**(mg)**	**(μL)**	**(μL)**
PPP	90[a]	0	0	0	0	40
PPP+Citrate	90[a]	0	10	0	0	30
PPP+MS@D-HMP+Ca^{2+}	90[a]	10	0	2.25	0	30
PPP+MS@D-HMP+Citrate	90[a]	0	10	2.25	0	30
PPP+Actin	90[a]	0	0	0	5	35
PPP+Actin+ Citrate	90[a]	0	10	0	5	25
dFXII PPP+Ca^{2+}	90[b]	5	0	0	0	35
dFXII PPP	90[b]	0	0	0	0	40
dFXII PPP+MS@D-HMP+Ca^{2+}	90[b]	10	0	2.25	0	30
dFXII PPP+MS@D-HMP	90[b]	0	0	2.25	0	40
dFXII PPP+Ca^{2+}+Actin	90[b]	10	0	0	5	25

Supplementary Table 9. Results of blood count assay *in vitro*. All the values are expressed as mean ± SD, n = 6 biologically independent samples. *WBC, white blood cell count; #NEUT, neutrophil count; #LYMPH, lymphocyte count; #MONO, monocyte count; #EOS eosinophil count; #BASO, basophil count; %NEUT, neutrophil ratio; %LYMPH, lymphocyte ratio; %MONO, monocyte ratio; %EOS, eosinophil ratio; %BASO, basophil ratio; RBC, red blood cell count; HGB, haemoglobin; HCT, haematocrit; MCV, mean corpuscular volume; MCH, mean corpuscular haemoglobin; MCHC, mean corpuscular haemoglobin concentration; RDW-CV, red blood cell distribution width-coefficient of variation; PLT, platelet; MPV, mean platelet volume. All the values are expressed as mean ± SD, n = 3 biologically independent samples.

Tests	Blood	MS	MS@D	MS@D-HMP
WBC (10^9/L)	7.96±0.48	4.50±0.61	5.02±0.65	4.69±0.26
#NEUT (10^9/L)	4.27±0.25	2.07±0.24	2.34±0.28	2.21±0.13
#LYMPH (10^9/L)	2.89±0.18	1.91±0.32	2.07±0.31	1.93±0.12
#MONO (10^9/L)	0.39±0.06	0.34±0.05	0.39±0.03	0.34±0.02
#EOS (10^9/L)	0.35±0.05	0.16±0.03	0.20±0.05	0.18±0.03
#BASO (10^9/L)	0.06±0.04	0.02±0.01	0.03±0.01	0.03±0.01
%NEUT (%)	53.6±1.2	46.0±1.6	46.7±1.7	47.1±1.2
%LYMPH (%)	36.4±1.1	42.3±1.8	41.1±1.3	41.2±1.5
%MONO (%)	4.9±0.6	7.5±0.5	7.7±0.6	7.3±0.3
%EOS (%)	4.4±0.5	3.7±0.3	3.9±0.7	3.9±0.5
%BASO (%)	0.8±0.5	1.5±2.0	0.6±0.1	0.6±0.1
RBC (10^{12}/L)	5.25±0.61	4.60±1.21	5.08±0.61	4.95±0.37
HGB (g/L)	179±21	168±17	163±21	159±14
HCT (%)	49.8±5.9	47.5±5.3	47.4±5.9	46.0±3.5
MCV (fL)	94.9±0.2	93.0±0.4	93.3±0.3	93.1±0.2
MCH (pg)	34.1±0.3	32.9±0.5	32.1±1.2	32.2±1.2
MCHC (g/L)	360±3	353±6	344±13	346±13
RDW-CV (%)	11.2±0.0	10.6±0.2	10.6±0.1	10.6±0.1
PLT (10^9/L)	345±47	159±11	172±11	171±20
MPV (fL)	9.7±0.3	9.7±0.2	9.8±0.3	9.8±0.1

Supplementary Table 10. Complete blood count assay for the transfused rabbit treated with heparin or MS@D-HMP at different time intervals. All the values are expressed as mean ± SD, n = 3 biologically independent samples.

**Tests**	**Control**		**1d**		**5d**	
	**Heparin**	**MS@D-HMP**	**Heparin**	**MS@D-HMP**	**Heparin**	**MS@D-HMP**
WBC (10^9/L)	8.53±1.87	4.50±1.66	18.74±3.50	12.71±1.31	10.19±2.04	7.95±1.94
#NEUT (10^9/L)	5.50±2.39	2.39±0.83	13.36±1.93	7.82±2.62	4.73±0.29	3.98±0.59
#LYMPH (10^9/L)	2.44±0.91	1.40±1.37	4.24±2.35	3.95±1.38	4.59±2.11	3.18±1.66
#MONO (10^9/L)	0.50±0.21	0.45±0.33	1.01±0.23	0.40±0.28	0.79±0.08	0.52±0.06
#EOS (10^9/L)	0.06±0.02	0.10±0.07	0.09±0.05	0.25±0.15	0.05±0.02	0.06±0.02
#BASO (10^9/L)	0.02±0.01	0.14±0.17	0.04±0.00	0.15±0.21	0.03±0.02	0.13±0.10
%NEUT (%)	61.1±18.6	57.0±16.7	72.6±9.2	60.2±16.6	48.3±10.1	52.3±12.6
%LYMPH (%)	32.3±19.3	28.5±19.7	21.3±10.3	32.4±14.5	42.9±10.7	37.7±13.3
%MONO (%)	5.6±1.2	9.4±6.5	5.5±1.2	3.0±1.8	7.9±0.8	6.9±2.0
%EOS (%)	0.7±0.2	2.3±1.4	0.4±0.2	1.9±0.9	0.5±0.1	0.9±0.4
%BASO (%)	0.3±0.2	2.5±2.5	0.2±0.1	1.3±1.9	0.3±0.1	1.5±0.8
RBC (10^{12}/L)	4.97±0.34	5.56±0.48	4.14±0.30	4.45±0.70	3.60±0.04	4.71±0.61
HGB (g/L)	112±10	125±4	92±11	101±10	78±7	103±9
HCT (%)	33.8±3.3	36.1±3.0	27.9±2.8	30.2±4.4	24.6±1.6	32.7±3.8
MCV (fL)	67.9±3.6	65.1±2.5	67.3±3.3	68.0±3.6	68.5±3.7	69.8±7.6
MCH (pg)	22.4±1.1	22.7±1.5	22.0±1.4	23.0±1.5	21.6±1.8	21.9±1.0
MCHC (g/L)	330±4	348±18	327±8	338±18	314±11	318±35
RDW-CV (%)	13.1±0.8	13.8±1.4	12.8±0.8	14.9±1.5	14.0±0.5	15.9±2.2
PLT (10^9/L)	309±115	296±81	304±84	341±75	476±94	563±27
MPV (fL)	6.3±0.8	7.2±0.8	6.3±0.5	6.6±0.2	6.3±0.4	6.5±0.9

Supplementary References

1. Ge J*, et al.* Joule-heated graphene-wrapped sponge enables fast clean-up of viscous crude-oil spill. *Nat. Nanotechnol.* **12**, 434-440 (2017).

2. Nishi Y, Dai GZ, Iwashita N, Sawada Y, Inagaki M. Evaluation of sorption behavior of heavy oil into exfoliated graphite by wicking test. *Mater. Sci. Res. Int.* **8**, 243-248 (2002).

3. Song X*, et al.* Transient blood thinning during extracorporeal blood purification via the inactivation of coagulation factors by hydrogel microspheres. *Nat. Biomed. Eng.* **5**, 1143-1156 (2021).
