# Supplementary material for: Self-anticoagulant sponge for whole blood auto-transfusion and its mechanism of coagulation factor inactivation
Source: Nat Commun. 2023 Aug 12;14:4875. doi: 10.1038/s41467-023-40646-7 (PMC10423252; doi:10.1038/s41467-023-40646-7)
Supplement: Supplementary file 3 — Description of Additional Supplementary Files Document [file 41467_2023_40646_MOESM3_ESM.pdf]

<u>Description of Additional Supplementary Files Document</u>

Supplementary Movie 1. Clotting behaviors of blood collected using MS@DHMP in the rabbit femoral artery hemorrhage model.

Supplementary Movie 2. Survival of rabbits one month after the whole blood auto-transfusion.

Supplementary Data 1

Relative abundance of top 10 most-abundant proteins identified from normal plasma and MS@D-HMP-treated plasma. Proteins were grouped according to biological processes of the blood system, and then ranked by abundance from highest to lowest. Relative abundance of proteins in different plasma was given to facilitate the comparison of difference. For unique protein existing in each sample, the corresponding relative abundance in another sample, if detected (but not in top 10 most-abundant), was provided. NA, no detection; a, relative abundance of proteins in MS@D-HMP-treated plasma; b, relative abundance of proteins in normal plasma.

Supplementary Data 2

Relative abundance of top 10 most-abundant tightly-bound proteins identified at stage 1 and 2. Proteins were grouped according to biological processes of the blood system, and then ranked by abundance from highest to lowest. Relative abundance of proteins at two stages was given to facilitate the comparison of difference. For unique protein existing in each sample, the corresponding relative abundance in another sample, if detected (but not in top 10 most-abundant), was provided. a, relative abundance of corona proteins in stage 2; b, relative abundance of corona proteins in stage 1.

Supplementary Data 3

Biological parameters levels for the transfused rabbit treated with heparin or MS@D-HMP at different time intervals. *ALP, alkaline phosphatase; ALT, alanine aminotransferase; AST, aspartate aminotransferase; GGT, gamma-glutamyl transferase; UREA, urea; CREA, serum creatinine; TP, total protein; ALB, albumin; GLB, globulin; A/G, albumin globulin ratio; C4, complement component 4; C3, complement component 3; IGA, immunoglobulin A; IGG, immunoglobulin G; IGM, immunoglobulin M; GLU, glucose; CHOL, cholesterol; TG, triglyceride; K, serum potassium; Na, serum sodium; Cl, serum chlorine; P, serum phosphorus; UA, uric acid; HDL, high-density lipoprotein; LDL, low-density lipoprotein; CA, serum calcium; DBIL, direct bilirubin; TBIL, total bilirubin. All the values are expressed as mean ± SD, n = 3 biologically independent samples.
