# Supplementary material for: Self-anticoagulant sponge for whole blood auto-transfusion and its mechanism of coagulation factor inactivation
Source: Nat Commun. 2023 Aug 12;14:4875. doi: 10.1038/s41467-023-40646-7 (PMC10423252; doi:10.1038/s41467-023-40646-7)
Supplement: Supplementary file 7 — Reporting Summary [file 41467_2023_40646_MOESM7_ESM.pdf]

nature portfolio

Corresponding author(s): Haifeng Ji, Weifeng Zhao

Last updated by author(s): Aug 2, 2023

Reporting Summary

Nature Portfolio wishes to improve the reproducibility of the work that we publish. This form provides structure for consistency and transparency in reporting. For further information on Nature Portfolio policies, see our Editorial Policies and the Editorial Policy Checklist.

Statistics

For all statistical analyses, confirm that the following items are present in the figure legend, table legend, main text, or Methods section.

n/a	Confirmed	
☐	☒	The exact sample size (*n*) for each experimental group/condition, given as a discrete number and unit of measurement
☐	☒	A statement on whether measurements were taken from distinct samples or whether the same sample was measured repeatedly
☐	☒	The statistical test(s) used AND whether they are one- or two-sided<br>*Only common tests should be described solely by name; describe more complex techniques in the Methods section.*
☒	☐	A description of all covariates tested
☒	☐	A description of any assumptions or corrections, such as tests of normality and adjustment for multiple comparisons
☐	☒	A full description of the statistical parameters including central tendency (e.g. means) or other basic estimates (e.g. regression coefficient) AND variation (e.g. standard deviation) or associated estimates of uncertainty (e.g. confidence intervals)
☐	☒	For null hypothesis testing, the test statistic (e.g. *F*, *t*, *r*) with confidence intervals, effect sizes, degrees of freedom and *P* value noted<br>*Give P values as exact values whenever suitable.*
☒	☐	For Bayesian analysis, information on the choice of priors and Markov chain Monte Carlo settings
☒	☐	For hierarchical and complex designs, identification of the appropriate level for tests and full reporting of outcomes
☒	☐	Estimates of effect sizes (e.g. Cohen's *d*, Pearson's *r*), indicating how they were calculated

Our web collection on statistics for biologists contains articles on many of the points above.

Software and code

Policy information about availability of computer code

Data collection	OneAttension 2.7 was used to collect the data of permeating behaviors of one water droplet on the samples. BD FACSDiva™ was used to acquire Flow cytometry profiles.
Data analysis	Microsoft Excel 2019, OriginPro 2018, Prism Graphpad 8.0, OMNIC 8, Avantage 5.967, TGA Analytical Software 4.2.3, and FlowJo 10.6.2 were used. For the mass-spectrometry-based proteomics, the raw MS files were analyzed and searched against human SwissProt database on the basis of the species of the samples using MaxQuant 1.6.15.0. Chemdraw (version) for all chemical structures.

For manuscripts utilizing custom algorithms or software that are central to the research but not yet described in published literature, software must be made available to editors and reviewers. We strongly encourage code deposition in a community repository (e.g. GitHub). See the Nature Portfolio guidelines for submitting code & software for further information.

Data

Policy information about availability of data

All manuscripts must include a data availability statement. This statement should provide the following information, where applicable:

- Accession codes, unique identifiers, or web links for publicly available datasets
- A description of any restrictions on data availability
- For clinical datasets or third party data, please ensure that the statement adheres to our policy

All data are available within the main text and its Supplementary Information file. Mass spectra were searched against the human SwissProt database (20422

entries) concatenated with reverse decoy database (http://uniprot.org; proteome ID: UP000005640; release numbers: 2021_01/2021_01). Proteomic data, including raw data and search results have been deposited in the ProteomeXchange database with dataset identifier: PXD044005. The corresponding ProteomeXchange details are available at https://proteomecentral.proteomexchange.org/cgi/GetDataset?ID=PXD044005. Source data are provided with this paper.

Human research participants

Policy information about studies involving human research participants and Sex and Gender in Research.

Reporting on sex and gender	All blood samples were collected from healthy male donors, and gender differences were not considered. Sex specific analysis was also not performed in this study. We used only blood from male donors in our study based on previous studies using blood from male donors (Song et al. Nat. Biomed. Eng. 2021, 5, 1143–1156. https://doi.org/10.1038/s41551-020-00673-x.). We believe that the difference of blood coagulation system between healthy male and female is negligible.
Population characteristics	Human fresh blood samples were collected from three 24-year-old healthy male donors without medical history.
Recruitment	The West China Hospital announced the recruitment of human research participants. These volunteers were recruited by voluntary registration and disease-history screening. The participants were randomly recruited and there was no direct relationship between the study team members and the participants. We excluded participants with low hemoglobin level, blood cells, and coagulation disorders, for example, anemia, hemophilia, thalassemia, sickle cell anemia, anemic diseases, as well as, participants taking medication that can interfere with blood cell functions such as Aspirin, and Ibuprofen.
Ethics oversight	The experiments were approved and performed by West China Hospital, Sichuan University, and all the experiments were performed in compliance with the relevant laws and national guidelines (GB/T 16886.4-2003/ISO 10993-4:2002, General Administration of Quality Supervision, Inspection and Quarantine of the People's Republic of China, Standardization Administration of the People's Republic of China). The procedure for collecting blood from human volunteers has been approved by the Institute of Blood Transfusion, Chinese Academy of Medical Sciences' Institutional Review Board (IRB, Ethics approval number: No.202024) with written consent from donors.

Note that full information on the approval of the study protocol must also be provided in the manuscript.

Field-specific reporting

Please select the one below that is the best fit for your research. If you are not sure, read the appropriate sections before making your selection.

☒ Life sciences ☐ Behavioural & social sciences ☐ Ecological, evolutionary & environmental sciences

For a reference copy of the document with all sections, see nature.com/documents/nr-reporting-summary-flat.pdf

Life sciences study design

All studies must disclose on these points even when the disclosure is negative.

Sample size	For in vitro experiments, sample sizes (N=3 minimum) were chosen based on preliminary experiments and previous laboratory experience from Changsheng and Kizhakkedathu laboratories (Song et al. Nat. Biomed. Eng. 2021, 5, 1143–1156. https://doi.org/10.1038/s41551-020-00673-x). For in vivo experiments, sample sizes were chosen based on preliminary experiments and prior publications (Zhuang et al. Adv. Mater. 2018, 30, 1804693. https://doi.org/10.1002/adma.201804693). As this proof-of-concept study involved the initial demonstration of new methodology, sample sizes were selected to ensure confidence in the reproducibility of the methodology as well as statistically testable results. An N of 3 animals was used for each condition in each experiment to ensure reproducibility.
Data exclusions	No data was excluded in the analysis.
Replication	Unless otherwise mentioned, at least 3 replications were performed for all the tests. All our attempts at replication were successful with similar results.
Randomization	For in vitro tests, all samples were allocated randomly into experimental groups. For in vivo tests, all New Zealand rabbits were randomly allocated into appropriate groups.
Blinding	The establishment of trauma-induced hemorrhage for rabbits was performed pairwise by 2 independent surgeons. The surgeons are blinded to the whole blood salvaged by the sponge during the surgery. During in vivo follow-up, the technicians acquiring data (that is, blood count assays, clotting time tests, biological parameters level assays, titration experiments for coagulation factors, and concentrations of FDP and D-dimer) were also blinded to the groups. Characterizations of the sponges (including energy dispersive spectra, elemental analysis, X-ray photoelectron spectroscopy, Fourier transform infrared spectroscopy, thermogravimetric analysis, mercury intrusion curves and permeating behaviors), in vitro anticoagulant studies (clotting time tests, plasma recalcification times, thromboelastography, thrombin generation assay), mechanistic exploration of depletion of coagulation factors (detection of the activities of coagulation factors, proteomic identification of proteins in plasma, equilibrium binding capacity and the corresponding inactivation efficiency of FXI, identification of tightly-bound proteins on the surface of sponges, evaluation of activation of kallikrein/kinin system, chromogenic assays, detection of the procoagulant behaviors of FXI via fluorescence-based FIXa activity assay, and correction assays), hemocompatibilities (including evaluation of complement activation, platelet adhesion and platelet activation, monocyte activation, red blood cell morphology and haemolysis, blood count assay in vitro) were all

conducted by an independent operator. The operator was blinded to group allocation during data collection and/or analysis. During data analysis, in particular, for image-based data analysis, at least two blinded graders performed the grading /evaluation (such as SEMs).

Reporting for specific materials, systems and methods

We require information from authors about some types of materials, experimental systems and methods used in many studies. Here, indicate whether each material, system or method listed is relevant to your study. If you are not sure if a list item applies to your research, read the appropriate section before selecting a response.

Materials & experimental systems

n/a	Involved in the study
☐	☒ Antibodies
☒	☐ Eukaryotic cell lines
☒	☐ Palaeontology and archaeology
☐	☒ Animals and other organisms
☒	☐ Clinical data
☒	☐ Dual use research of concern

Methods

n/a	Involved in the study
☒	☐ ChIP-seq
☐	☒ Flow cytometry
☒	☐ MRI-based neuroimaging

Antibodies

Antibodies used

For ELISA methods, antibody-coated wells were provided by ELISA kit, and used based on the manufacturer's instruction. The antibody-coated well from Human Thrombin-Antithrombin Complex AssayMax kit was purchased from Assay Pro, USA. The antibody-coated wells from Human Bradykinin Enzyme Immunoassay Kit and Human Platelet Factor 4 Kit were purchased from RayBiotech Life, Inc., USA. The antibody-coated wells from Complement C3a Human ELISA Kit and Complement C5a Human ELISA Kit were purchased from Thermo Scientific, USA. The antibody-coated well from Rabbit D-dimer ELISA Kit was purchased from Zeye Biotech, China. The antibody from the Fluorescence-based Factor IXa Activity Assay Kit was purchased from BioVision Inc., USA. The antibody-coated wells from Human D-dimer ELISA Kit was purchased from Cusabio, China.

For FACS, anti-CD41a-FITC from eBioscience™, #11-0419-42, clone HIP8, dilution: 1 in 18. Anti-CD62p-APC from eBioscience™, #17-0626-82, clone Psel.KO2.3, dilution: 1 in 72. Anti-CD14-FITC from eBioscience™, #11-0149-42, clone 61D3, dilution: 1 in 20. Anti-CD11b-APC from eBioscience™, #17-0118-42, clone ICRF44, dilution: 1 in 20.

Validation

For ELISA, the antibodies were validated by the vendors; further validation was performed by positive control samples.

For FACS, antibodies were validated by comparison with any negative control sample. The validation of antibodies was also previously reported (anti-CD41a-FITC: Tuong et al, Cell Rep. 2021, 37(12), 110132. https://doi.org/10.1016/j.celrep.2021.110132. Anti-CD62p-APC: Liu et al, Cell Res. 2018, 28(9), 918-933. https://doi.org/10.1038/s41422-018-0070-2. Anti-CD14-FITC: Jiang et al, Cell Death Dis. 2022, 13(2), 183. https://doi.org/10.1038/s41419-022-04640-z. Anti-CD11b-APC: Lai et al, J. Inflamm. Res. 2021, 14, 5241-5249. https://doi.org/10.2147/JIR.S329921)

Animals and other research organisms

Policy information about studies involving animals; ARRIVE guidelines recommended for reporting animal research, and Sex and Gender in Research

Laboratory animals

Healthy New Zealand White Rabbits (14-16 weeks, male, about 3-4 kg of weight, Laboratory Animal Center of West China Hospital Science Park, Sichuan University).

Wild animals

The study did not involve wild animals.

Reporting on sex

All male animals were used in this study, and gender differences were not considered. We used only male animals in our study because methods to establish hemorrhage models in this study have been commonly reported in previous studies using either male animals or female animals or both (He et al. Nat. Commun. 2022, 13(1), 552. https://doi.org/10.1038/s41467-022-28209-8. Gao et al. Sci. Adv. 2020, 6(31), eaba0588. https://doi.org/10.1126/sciadv.aba0588).

Field-collected samples

The study did not involve samples collected from the field.

Ethics oversight

All procedures involving the use of animals in this study were prospectively reviewed and approved by the Institutional Animal Care and Use Committee. This study was conducted in accordance with the National Institutes of Health Guide for the care and use of laboratory animals (NIH Publications No. 8023, revised 1978). This experiment conformed to the legal requirement in China and was approved by the ethical committee (No. 2021911A) of West China Hospital, Sichuan University.

Note that full information on the approval of the study protocol must also be provided in the manuscript.

Flow Cytometry

Plots

Confirm that:

☒ The axis labels state the marker and fluorochrome used (e.g. CD4-FITC).

☒ The axis scales are clearly visible. Include numbers along axes only for bottom left plot of group (a 'group' is an analysis of identical markers).

☒ All plots are contour plots with outliers or pseudocolor plots.

☒ A numerical value for number of cells or percentage (with statistics) is provided.

Methodology

Sample preparation	Human platelet and whole blood were used for analysis. Details are given in the supplementary information.
Instrument	Flow cytometry profiles were acquired using a Multicolor Flow Cytometer (BD FACSCelesta™).
Software	All experimental acquisition was performed using BD FACSDiva™. FlowJo 10.6.2 software (TreeStar Inc) was used for data analysis.
Cell population abundance	A total of 10,000 events were collected for each sample.
Gating strategy	An illustration of the gating strategy used for flow cytometry analysis is given in the Supplementary Figure 28.

☒ Tick this box to confirm that a figure exemplifying the gating strategy is provided in the Supplementary Information.
